# Apelin and Apelin Receptor in Follicular Granulosa Cells of Buffalo Ovaries: Expression and Regulation of Steroidogenesis

**DOI:** 10.3389/fendo.2022.844360

**Published:** 2022-03-10

**Authors:** Borhan Shokrollahi, Hai-Ying Zheng, Ling-Yu Li, Li-Ping Tang, Xiao-Ya Ma, Xing-Rong Lu, An-Qin Duan, Yu Zhang, Xiao-Hui Tan, Chen-Xi Huang, Yuan-Yuan Xu, Jiang-Hua Shang

**Affiliations:** ^1^ Key Laboratory of Buffalo Genetics, Breeding and Reproduction Technology, Guangxi Buffalo Research Institute, Chinese Academy of Agricultural Sciences, Nanning, China; ^2^ Department of Animal Science, Sanandaj Branch, Islamic Azad University, Sanandaj, Iran

**Keywords:** apelin, apelin receptor, estradiol, progesterone, granulosa cells, buffalo

## Abstract

Apelin (APLN), as a ligand for APJ (an orphan G-protein-coupled receptor), is an adipokine with pleiotropic effects in many physiological processes of the body. It has an important role in the control of reproduction particularly in females (mainly in control of ovarian function). This study was carried out to investigate the mRNA and protein amounts of APLN/APJ in granulose cells (GCs) of ovarian follicles with small (SF), medium (MF), and large (LF) sizes of buffalo (*Bubalus bubalis*) and the effect of IGF1 and follicle-stimulating hormone (FSH) on the expression levels of APLN/APJ. In addition, we evaluated the effect of various doses of APLN (isoforms -13 and -17) singly or in combination with IGF1 and FSH on estradiol (E2) and progesterone (P4) secretion in GCs. The mRNA and protein abundance of APLN was the highest in GCs of LF while the APJ expression enhanced with follicle enlargement in GCs (p-value <0.01). IGF1 and FSH elevated the mRNA and protein amounts of APLN and FSH, and IGF1 increased the expression of APJ in buffalo GCs (p-value <0.01). Both isoforms of APLN (-13/-17) singly or in the presence of IGF1 or FSH increased the secretion of E2 and P4 with or without preincubation of cells with APJ antagonist (ML221 10 µM), although we had some variation in the effects. Concurrently, APLN-13/-17 significantly increased the mRNA and protein expression of CYP19A1 and StAR (p-value <0.01). ML221 substantially diminished the secretion of E2 and P4 and also the expression of CY19A1 and StAR in buffalo GCs (p-value <0.01). We also revealed that APLN-13/-17 (10^-9^ M), singly or in response to IGF1 and FSH, increased the production of E2 and P4 in different times of stimulation. In conclusion, APLN may play a crucial role in steroidogenesis and follicular development in ovarian GCs of buffalo.

## Introduction

As the largest endocrine gland, adipose tissue secretes different bioactive peptides that are mainly named adipokines (1). Apelin (APLN) is a new adipokine derived from preproapelin with 77 amino acids. Then, a 55-amino-acid fragment is derived from preproapelin and, subsequently, smaller bioactive isoforms such as APLN-36, APLN-17, and APLN-13 as well as the pyroglutamyl form of APLN -13 are produced [(Pyr-APLN-13; (2)]. The smaller isoforms ((Pyr) APLN-13 and -17) are more active and frequent in the circulation (3). APLN is initially detected in bovine stomach extracts as an internal ligand for APJ (an orphan G protein-coupled receptor) that is analogous to the angiotensin II type 1 receptor [AT1; (4)]. APJ can connect to various APLN isoforms with different affinities and switch on numerous signaling pathways resulting in divergent consequences in the body (5). In addition to adipose tissue, both APLN and APJ are extensively spread in the body and produced at different abundances in almost all tissues, particularly in the brain, heart, blood vessels, spleen, lung, intestine, breast, and reproductive tract (5–7). Furthermore, APLN and APJ are involved in several different basic biological processes, such as cell proliferation, cardiovascular function, angiogenesis, food intake, fluid homeostasis, and regulation of energy metabolism (5, 8–10). They have also been detected in the reproductive organs as well as in nucleus of the hypothalamus (the arcuate, supraoptic, and paraventricular hypothalamic), addressing their key roles in the regulation of reproduction (11). Shimizu etal. (12) showed that APJ is expressed in the granulosa cells (GCs) of the bovine follicles. They also suggested that APLN and APJ are partly responsible for the selection and dominance of follicles in the bovine species. The steroidogenic roles of APLN and APJ have been reported in different species such as rats, humans, cattle, sheep, and pigs (13–17). In cattle GCs, APJ but not APLN mRNA was expressed (12). Moreover, APLN and APJ were immunolocalized in porcine GCs and their expression were rather than theca cells (15). Furthermore, APLN and APJ expression was detected in large luteal cells in addition to GCs in sheep (17). Some literature showed that APLN singly or with IGF1 and FSH has some effects on the secretion of progesterone (P4) in cattle (16) and porcine (15). It exerts its effects through various signaling pathways. APLN also led to an increase of the basal levels of estrogen (E2) and P4 *via* AMPKα activation and enhanced the concentration of HSD3B protein, and it decreased the IGF1- and FSH-induced steroid secretion in human and porcine GCs (14–16).

The literature shows that ovarian factors can influence the expressions of APLN and APJ. For instance, IGF1 increased the APLN expression; however, it diminished the mRNA expression of APJ (16). P4 and FSH enhanced the expression of APJ in bovine GCs, while LH affected the expression of both APLN and APJ in cultured TCs (12). E2 causes the gene expressions of APLN and APJ in follicles, indicating the correlation of these genes with angiogenesis during follicle maturation and corpus luteum development in the bovine ovary. APLN and APJ have been involved in the atresia of follicles, the temporary luteal phase after ovulation, and the luteolysis process of the corpus luteum (13). In addition, elevated levels of APJ in atretic follicles were correlated with follicular atresia originating from GC apoptosis in bovine (12). In addition, Roche etal. (16) revealed that APLN and its receptor are expressed in GCs and oocytes in cattle.

Nevertheless, the expression and the possible roles of the APLN and APJ system have not been studied in the GCs of buffalo ovaries. Accordingly, the current study was aimed to investigate the expression of APLN and APJ in GCs of ovarian follicles and the *in vitro* effects of APLN on E2 and P4 production along with the associated molecular mechanisms in ovarian GCs of buffalos.

## Materials and Methods

### Reagents and Ethical Statement

All chemicals and media used in the current study were obtained from Sigma-Aldrich (St. Louis, MO, USA) if not otherwise specified. Total approaches of experiments were performed by the Animal Ethics Committee of the Guangxi Buffalo Research Institute and were implemented with the ethical regulations of animal research by this committee.

### Hormones and Antibodies

Recombinant porcine FSH, recombinant human IGF1 (ab270062), APLN-13 (ab141010), and APLN-17 (ab141011) were purchased from Abcam. Also, APLN (ab141011), CYP19A1 (ab18995), StAR (ab96637), and actin (ab8226) antibodies were obtained from Abcam. APJ (20341-1-AP), anti-mouse IgG (SA00001-1), and anti-rabbit IgG (SA00001-2) were obtained from Proteintech (Wuhan, China). Primary and secondary antibodies were used at 1: 500 to 1:1,000 and 1/3,000 for Western blotting, respectively.

### Follicle Collection and Granulosa Cell Culture

The obtained buffalo ovaries from a regional slaughterhouse were transferred to the laboratory on ice within 2 h after slaughter. The buffalos were originated from different farms in Guangxi province, China. During transportation, the ovaries were kept in phosphate-buffered saline (PBS) containing 0.06 mg/ml penicillin and 0.05 mg/ml streptomycin at nearly 30°C–35°C. In the laboratory, the ovaries were properly washed with physiological saline solution.

To evaluate the effect of the APLN on follicle steroidogenesis, a GC culture model was established. Therefore, all the intact (adequate circularized with clear follicular fluid and, wall) and detectable follicles were extracted by a 17-gauge needle linked to a 10-ml syringe. The extracts with PBS were moved to a 60-mm dish in a sterilized environment, and all cumulus–oocyte complexes were taken out. The residuals were centrifuged in 15-ml conical tubes at 700 g for 5 min. Then, GCs were resuspended in Dulbecco’s modified Eagle medium (DMEM) medium consisting of 10% fetal bovine serum (FBS) and a solution of antibiotics and antimycotic (penicillin 100 U/ml, streptomycin 100 mg/ml, amphotericin B 0.25 mg/ml). Trypan blue exclusion dye was used for assessment of cell viability and count which was higher than 80%. Afterward, the cells were cultivated in a 24/48-well plate at 37.5°C in a humidified CO_2_ (5%) incubator and included relatively 1.5 × 105 live cells per well. Following the attachment and growth of cells with about 75%–80% confluence) for 48 h, they were supplemented with fresh media (FBS free) having various concentrations (10^-9^, 10^-8^, and 10^-6^ M) of recombinant APLN-13 or -17 singly or at the concentration of 10^-9^ with porcine FSH (10^-8^ M) or human recombinant IGF-I (10^-8^ M) and were kept for 48 h. Control cells were multiplied in the same circumstances as other cells except for the addition of the peptides. Six replicates were tested for each experimental condition. The spent media were accumulated after 48 h and reserved for E2 and P4 assay, and RNA and protein were extracted from cells.

To compare APLN and APJ expression in different groups of follicles, they were assigned into three different classes according to diameter (3–5 mm, small (SF); 6–9 mm, medium (MF); and >9 mm, large (LF)). In each group of follicles, GCs were collected for mRNA and protein extraction and promptly frozen in liquid nitrogen and stored at -80°C.

### Total RNA Extraction, cDNA Synthesis

Total RNA was extracted from GCs of follicles using TRIZOL reagent by the manufacturer’s instruction and a fixed quantity of RNA (100 ng) was straightly reverse-transcribed into a 20-μl first-strand cDNA using a PrimeScript RT reagent Kit with gDNA Eraser (Perfect Real Time, Takara Bio Inc., Shiga, Japan) according to the manufacturer’s instructions.

### Quantitative Real-Time PCR Analysis

Rt-qPCR was done in a whole amount of 20 μl, having the same disseminated cDNA (100 ng), 10 mM each of the forward and reverse primers, and 10 μl of 2× SYBR Green Master Mix (SYBR^®^ Premix Ex Taq™ II (Tli RNaseH Plus, Takara, Japan). The reactions were done in triplicate for all genes of interest and were run on the LightCycler 480 system (Roche Diagnostics, Basel, Switzerland) under the subsequent conditions: 95°C for 30 s, followed by 40 cycles at 95°C for 5 s and 60°C for 30 s. β-Actin and RPS15 were adopted as the internal control (reference genes) to normalize the relative gene expression levels. All reactions were carried out in triplicate. The expression abundances of genes were evaluated with the 2^−ΔΔCT^ method described previously by Livak and Schmittgen (18) concerning the housekeeping genes. The specifications of the genes of interest and the primer pairs used in the study are provided in **Table 1**.

**Table 1:** Gene, primer sequence (5′–3′), and developing fragment size.

Gene	Primer sequence (5′–3′)	Amplicon size (bp)	Accession no.
β-Actin	F: TCTCACGGAGCGTGGCTACAG R: CTGCTCGAAGTCCAGGGCCACGTA	100	NM_001290932.1
RPL15	F: TGGGCTACAAGGCCAAACAA R: GCTTCGAGCAAA CTTGAGCTGG	140	MG969348
APLN	F: AAGGCACCATCCGATACCTG R: ATGGGACCCTTGTGGGAGA	106	(16)
APJ	F: TCTGGGCCACCTACACCTAT R: ACGCTGGCGTACATGTTG	100	(16)
CYP19A1	F: CGTCCTGGTCACCCTTCT R: ACGCACCGACCTTGCAA	57	(19)
StAR	F: CTGCGTGGATTAACCAGGTTCG R: CCAGCTCTTGGTCGCTGTAGAG	84	(19)

### Western Blot Analysis

Total proteins were obtained from cultured GCs of different experiments by lysing in RIPA buffer with PMSF (R0010; Solarbio, Beijing, China) at 4°C for 30 min followed by collection and centrifugation at 12,000 rpm for 5 min at 4°C. The pellet was eliminated, and lysates were mitigated with 6× protein loading buffer (DL101-02; TransGen, Beijing, China) and heated to 100°C for 5 min. Following chilling at refrigerator temperature, the samples were stored at -80°C for Western blotting on ice afterward. Western blotting was started by loading the samples on a 12% gradient polyacrylamide gel (P0012AC; Beyotime, Shanghai, China) and then handing it over to a PVDF membrane (ISEQ00011; Millipore, Shanghai, China), followed by blocking in 8% (wt/vol) Difco Skim Milk in Tris-buffered saline including 0.1% (vol/vol) Tween-20 (TBST) for 2 h. The membrane was incubated with monoclonal anti-β-catenin antibody, diluted 1:500 in blocking buffer, whereas, as the molecular weights of proteins (APLN with 9 kDa, APJ with 68 kDa, StAR with 32 kDa, and CYP19A1 with 58 kDa) and β-actin (43 kDa) were different, each half was marked with a separate primary antibody. For other proteins, the membrane was maintained with their primary antibodies, diluted 1:500 to 1:1,000 in blocking buffer, and incubated overnight at 4°C. After four washes, 10 min each, with TBST, membranes were incubated for 1 h at 37°C with goat anti-mouse IgG (SA00001-1, Proteintech, China) for beta-actin and APLN, APJ, StAR, and CYP19A1 with goat anti-rabbit IgG (SA00001-2, Proteintech, China). The membranes were washed four more times in TBST for 10 min each, then developed using ECL Plus (P0018, Beyotime, China) followed by detection with a multifunction imager (Syngene, Cambridge, UK). The strength of each band was normalized to beta-actin expression. Western blots were conducted three times.

### Steroid ELISA Assay

Progesterone concentrations were estimated in serum-free medium from buffalo GCs after 48 h of culture using ELISA kits supplied by Miebiao Biology, China. Cells were plated in 24- or 48-well plates (10^5^ live cells/well), and six or eight replicates were examined for each tested condition (rh APLN-13/-17, IGF1, FSH) with or without (W/O) ML221 for each culture. The results were represented as the levels of the steroid (ng/mL). The intra- and inter-assay coefficients of variation (CV) for E2 and P4 were lower than 10%. Results are shown as mean ± SEM. Data were collected from three separate cultures.

### Statistical Analysis

**Figure 1:**
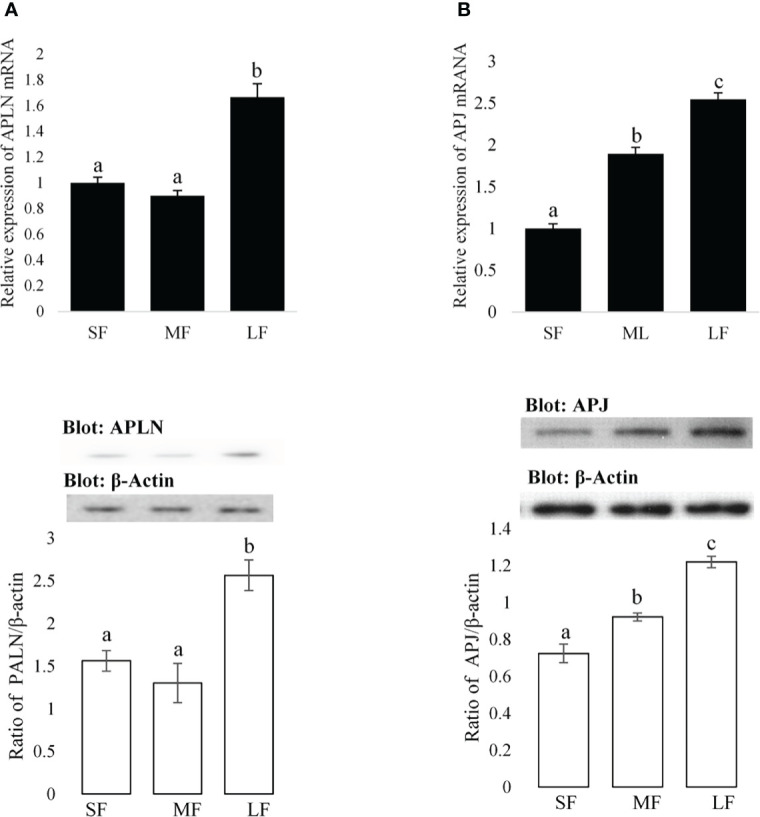
Expression amounts of the APLN and APJ in ovarian GCs of buffalo. Protein and mRNA expression of APLN **(A)** and APJ **(B)** was assessed by quantitative real-time PCR and Western blot in GCs from small (SF), medium (MF), and large (LF) follicles. β-Actin and RPL15 were used as housekeeping genes for qPCR and β-actin as a loading control for Western blot. A pool of 15 follicles for each group was used for the study of APLN and APJ protein and mRNA expression. Then, GC protein extracts were immunoblotted by APLN and APJ antibodies. The membranes were then reprobed by an anti-β-actin antibody with the same amount of protein loading for confirmation. By the evaluation of blots, the ratios of APLN and APJ to -β-actin were calculated. Statistical analyses were done for APLN and APJ, and the results are presented as the mean ± SEM. The different letters on the bars show significant differences at a p-value < 0.01.

**Figure 2:**
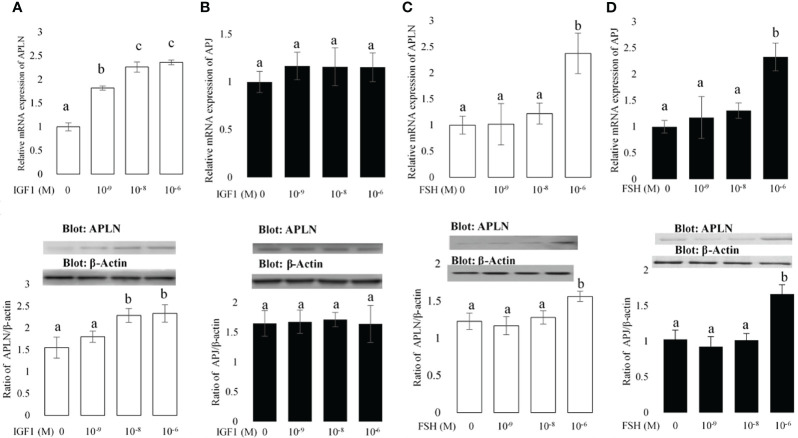
The effects of different concentrations of IGF1 and FSH on the mRNA and protein expression amounts of APLN **(A, C)** and APJ **(B, D)** in buffalo GCs. qPCR was used to assess APLN and APLJ mRNA expression in buffalo GCs. Fresh cells were seeded in a DMEM (with 10% FBS) and after 48 h; culture continues in free FBS media containing IGF1 or FSH with concentrations 0, 10^-9^, 10^-8^, and 10^-6^ M for an additional 48 h. GC protein extracts were immunoblotted by APLN and APJ antibodies. The membranes were then reprobed by an anti-β-actin antibody with the same amount of protein loading for confirmation. By the evaluation of blots, the ratios of APLN and APJ to -β-actin were calculated. Statistical analyses were done, and the results are presented as the mean ± SEM. The different letters on the bars show significant differences at a p-value < 0.05.

**Figure 3:**
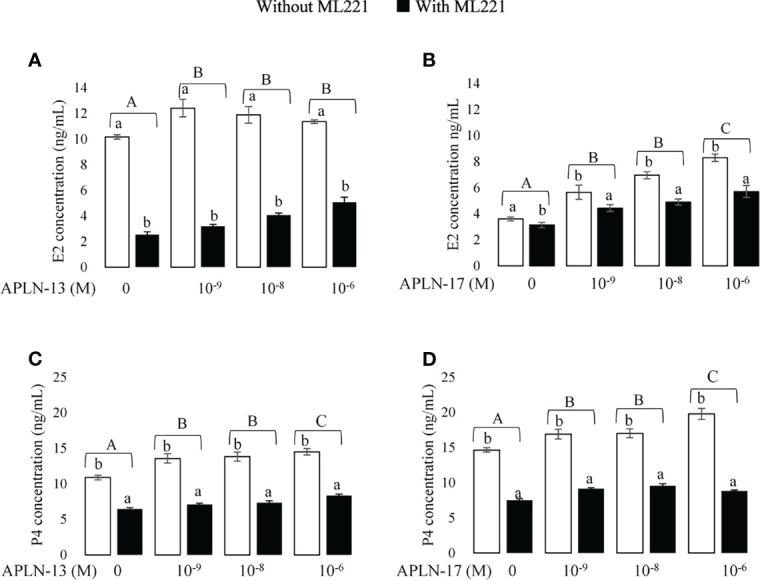
The effect of APLN-13/-17 on E2 and P4 synthesis in buffalo GCs. Cells were seeded in DMEM (with 10% FBS) for 48h in and then in free serum DMEM medium containing different concentrations of APLN-13/-17 (0, 10^-9^, 10^-8^, and 10^-6^ M) for 48 h in the presence or absence of APJ antagonist [ML221 (10 µM)]. After the collection, the culture medium was examined for E2 and P4 by ELISA. Results are means + SEM of 6 replicates. Different capital letters show a significant effect of treatments on E2 [APLN-13 **(A)**, APLN-17 **(B)**] and P4 [APLN-13 **(C)**, APLN-17 **(D)**], and lowercase letters indicate a significant effect of ML221 treatment. The different letters on the bars show significant differences at a p-value < 0.01.

**Figure 4:**
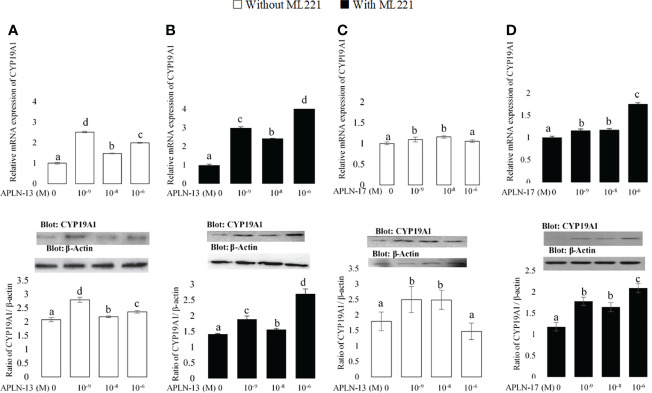
The protein and mRNA expression of CYP19A1 in the buffalo GCs treated by different doses of APLN-13/-17 without **(A, B)** or with **(C, D)** preincubation of cells with APJ antagonist [ML221 (10 µM)]. GCs were seeded in DMEM (with 10% FBS) for 48 h in and then in free serum DMEM medium containing different concentrations of APLN-13/-17 (0, 10^-9^, 10^-8^, and 10^-6^ M) for 48 h in the presence or absence of APJ antagonist [ML221 (10 µM)]. Following the total protein and mRNA extraction from the treated buffalo GCs, they were used to assess the expression levels of CYP19A1 by Western blot and quantitative real time-PCR, respectively. β-Actin and RPL15 expression levels in the same mRNA samples were used to normalize the mRNA expression of CYP19A1. The upper and lower parts of the panels show the relative levels of mRNA and illustrative immunoblots, respectively. In this regard, GC protein extracts were immunoblotted by the CYP19A1 antibody. The membranes were then reprobed by an anti-β-actin antibody with the same amount of protein loading for confirmation. By the evaluation of blots, the ratio of CYP19A1 to β-actin was calculated. Statistical analyses were done, and the results are presented as the mean ± SEM. The different letters on the bars show significant differences at a p-value < 0.05.

**Figure 5:**
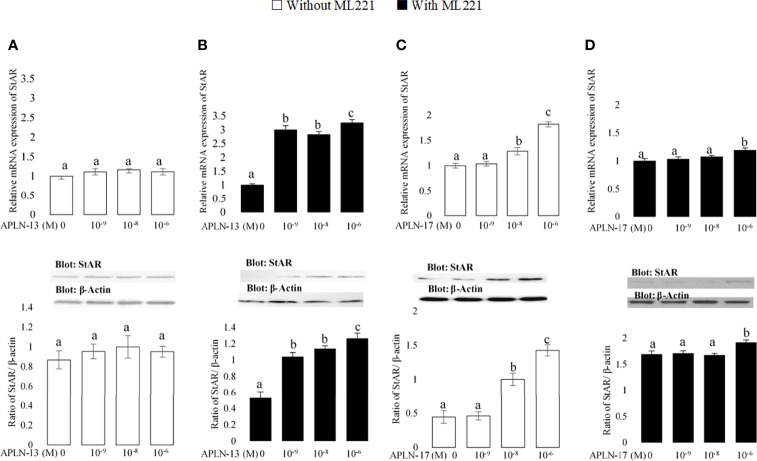
The protein and mRNA expression of StAR in the buffalo GCs treated by different doses of APLN-13/-17 without **(A, B)** or with **(C, D)** preincubation of cells with APJ antagonist (ML221 (10 µM)). GCs were seeded in DMEM (with 10% FBS) for 48 h in and then in free serum DMEM medium containing different concentrations of APLN-13/-17 (0, 10^-9^, 10^-8^, and 10^-6^ M) for 48 h in the presence or absence of APJ antagonist [ML221 (10 µM)]. Following the total protein and mRNA extraction from the treated buffalo GCs, they were used to assess the expression levels of StAR by Western blot and quantitative real time-PCR, respectively. β-Actin and RPL15 expression levels in the same mRNA samples were used to normalize the mRNA expression of StAR. The upper and lower parts of the panels show the relative levels of mRNA and illustrative immunoblots, respectively. In this regard, GC protein extracts were immunoblotted by the StAR antibody. The membranes were then reprobed by an anti-β-actin antibody with the same amount of protein loading for confirmation. By the evaluation of blots, the ratio of StAR to β-actin was calculated. Statistical analyses were done, and the results are presented as the mean ± SEM. The different letters on the bars show significant differences at a p-value < 0.05.

**Figure 6:**
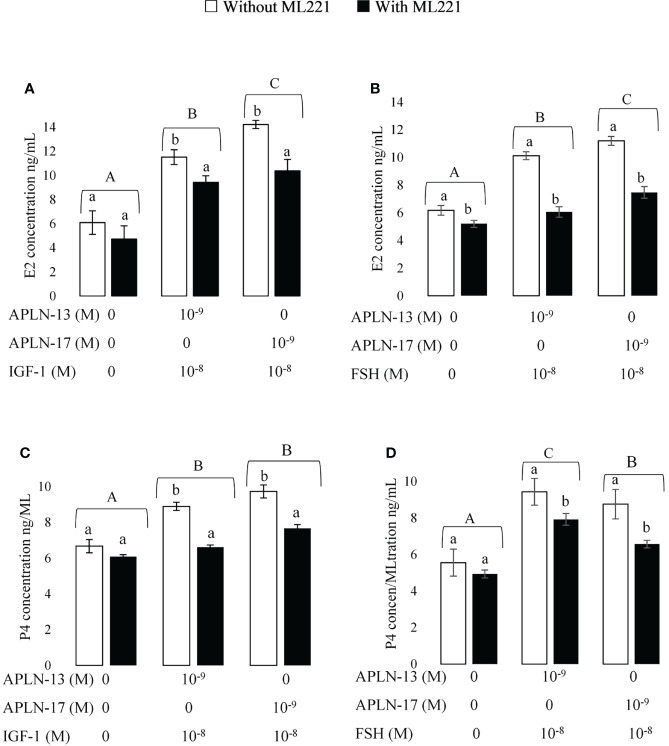
The concentration of E2 **(A,B)**; and P4 **(C,D)** in spent media of buffalo GC culture. GCs were seeded in DMEM (with 10% FBS) for 48 h in and then in free serum DMEM medium solely or containing APLN-13/-17 at the dose 10^-9^ M or in combination with IGF1 (10^-8^) or FSH (10^-8^) for 48 h in the presence or absence of ML221 (10 µM). After the collection, the culture medium was examined for E2 and P4 by ELISA. Results are means + SEM of 6 replicates. Different capital letters show a significant effect of treatments on E2 [APLN-13/-17 with IGF1 **(A)**, APLN-13/-17 with IGF1 **(B)**] and P4 [APLN-13/-17 with IGF1 **(C)**, APLN-13/-17 with IGF1 **(D)**] and lowercase letters indicate a significant effect of ML221 treatment.

**Figure 7:**
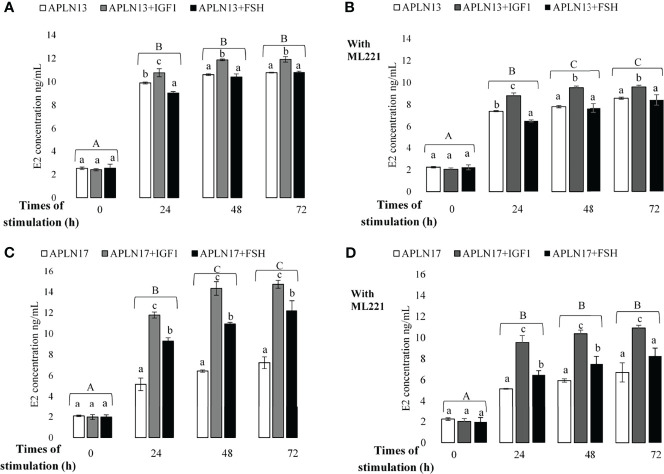
The concentration of E2 in buffalo GCs treated by APLN-13 (10^-9^; **A, B**) or APLN-17 (10^-9^; **C, D**) solely or in combination with IGF1 (10^-8^) and FSH (10^-8^) in different times of stimulation (0, 24, 48, and 72 h) with **(B, D)** or without **(A, C)** preincubation of cells with ML221 (10 µM). GCs were seeded in DMEM (with 10% FBS) for 48 h in and then in free serum DMEM medium according to the above description. After the collection, the culture medium was examined for E2 and P4 by ELISA. Different capital letters show a significant effect of different times of stimulation by treatments on E2 [without ML221 **(A)**, with ML221 **(B)**] and P4 [without ML221 **(C)**, with ML221 **(D)**] and lowercase letters indicate a significant effect of different treatment (APLN-13/-17 in the presence of IGF1 or FSH). Results are means ± SEM of 6 replicates. The different letters on the bars show significant differences at a p-value < 0.05.

**Figure 8:**
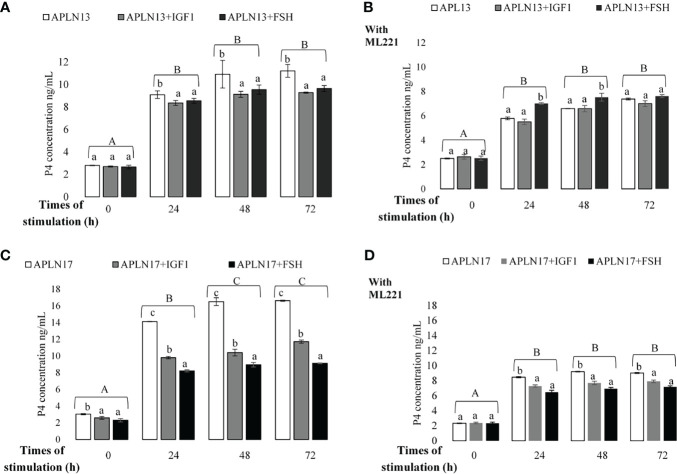
The concentration of P4 in buffalo GCs treated by APLN-13 (10^-9^; **A, B**) or APLN-17 (10^-9^; **C, D**) solely or in combination with IGF1 (10^-8^) and FSH (10^-8^) in different times of stimulation (0, 24, 48 and, 72 h) with **(B, D)** or without **(A, C)** preincubation of cells with ML221 (10 µM). GCs were seeded in DMEM (with 10% FBS) for 48 h in and then in free serum DMEM medium according to the above description. After the collection, the culture medium was examined for E2 and P4 by ELISA. Different capital letters show a significant effect of different times of stimulation by treatments on E2 (without ML221 **(A)**, with ML221 **(B)**) and P4 (without ML221 **(C)**, with ML221 **(D)**), and lowercase letters indicate a significant effect of different treatment (APLN-13/-17 in the presence of IG F1 or FSH). Results are means ± SEM of 6 replicates. The different letters on the bars show significant differences at a p-value < 0.05.

All the data have been shown as mean ± SEM. One-way (**Figures 1**, **2**, **4**, **5**) or two-way (**Figures 3**, **6**–**8**) ANOVA followed by Duncan’s multiple-range test was used to test the differences among groups by GLM procedure of SAS software (Version 9.4). A significant difference of p-value < 0.05 was taken into account. The data were evaluated for normality and homogeneity of variance among the treatments.

## Results

### Expression of APLN and APJ in the GCs of Follicles With Different Sizes

The expression levels of APLN and APJ in GCs of small (SF), medium (MF), and large (LF) size follicles are presented in **Figure 1**. To assess the relative mRNA expression levels of APLN and APJ in ovarian GCs, two cDNA fragments of 106 and 100 bp were amplified by qRT-PCR corresponding to APLN and APJ, respectively. The analysis of APLN transcript by qRT-PCR showed a varied expression level during the follicular phase. The mRNA expression of APLN significantly (p-value <0.01) increased in GCs of LFs compared to those of SFs or MFs. Its mRNA expression was not significantly different in GCs of Sf and MF, although its expression numerically declined in GCs of Mfs (**Figure 1A**). The expression of APJ in GCs significantly increased with an increase in the size of the follicles (**Figure 1B**; p-value <0.01). The APJ transcriptional abundance was the lowest in SFs and the highest in LFs. The presence of APLN and APJ proteins in GCs of different sizes of buffalo follicles was confirmed by Western blot. The immunoblot bands of APLN (9 kD), APJ (68 kD), and β-actin (42 kD) are evident in **Figure 1**. The protein expressions of APLN and APJ (ratio of optical density of APLN or APJ proteins/optical density of b-actin protein) in GCs were similar to the relative mRNA expressions.

### Effect of IGF1 and FSH on the Transcript Abundances of APLN and APJ in Buffalo GCs

The effects of IGF1 and FSH on APLN and APJ expression are shown in **Figure 2**. The effects of IGF1 and FSH as two major hormones influencing the multiplication and differentiation of ovarian cells at concentrations of 0, 10^-9^, 10^-8^, and 10^-6^ were studied on the expression of APLN and APJ in buffalo GCs. The results of qPCR showed that all doses of IGF1 enhanced the expression of APLN (**Figure 2A**); however, the Western blot results did not verify the effect of IGF1 at the dose of 10^-9^ on APLN level. Moreover, IGF1 did not have any effect on the mRNA or protein expression of APJ at all (**Figure 2A**). In addition, FSH at the concentration of 10^-6^ M (not other doses) increased the mRNA expression levels of APLN and APJ (**Figures 2C, D** **)**. These results were verified by immunoblotting of APLN and APJ proteins.

### Effect of APLN on E2 and P4 Production in Cultured Buffalo GCs

The concentrations of E2 and P4 in the spent media of GC culture are presented in **Figure 3**. With the aim of detecting the effect of APLN on the production of E2 and P4, buffalo GCs were cultured in the medium containing different levels of APLN-13/-17 (10^-9^, 10^-8^, and 10^-6^ M) for 48 h. Moreover, to verify a clear-cut effect of APLN, buffalo GCs were preincubated by the APJ antagonist, ML221 for an hour, in the same conditions outlined above. Results showed that APLN-13 (**Figures 3A, C** **)** and APLN-17 (**Figures 3B, D** **)** significantly increased the secretion of E2 and P4. ML221 (APJ antagonist) significantly diminished the secretion of E2 and P4 in buffalo GCs. Moreover, the effect of APLN-13/-17 on the levels of E2 and P4 was not the same in the presence or absence of ML221. Without ML221, APLN-13 had numerically a higher effect on the secretion of E2 at the concentration 10^-9^ M compared to concentrations of 10^-8^ and 10^-6^M. However, E2 increased by the treatment of APLN-13 or APLN-17 in the presence of ML221 in a dose-dependent manner. Furthermore, P4 concentration increased (p-value < 0.01) by the treatment of buffalo GCs with all concentrations of APLN-13 (W/O ML221). APLN-17 treatment at the concentrations of 10^-9^, 10^-8^, and 10^-6^ M enhanced the P4 secretion of GCs in the absence of ML221, but the same effects were not seen with ML221. The highest dose of APLN-17 (10^-6^ M) had the greatest effect on P4 secretion without ML221, but it was the lowest by preincubation of GCs with ML221 for an hour.

### Effects of APLN on the Transcription Amount of Some Factors Involved in E2 and P4 Production

The effects of APLN-13/-17 on the transcription amount of CYP19A1 as an important steroidogenesis enzyme as well as that of StAR as an important cholesterol transporter were evaluated to better understand their effect on steroidogenesis. As revealed in **Figure 4**, APLN-13 and APLN-17 W/O preincubation of GCs by ML221 for an hour increased the protein and mRNA levels of CYP19A1. Furthermore, APLN-13 (with ML221; **Figure 5B**) significantly increased the protein and mRNA amount of StAR at all treated concentrations but the same effect was not observed in the absence of ML221 (**Figure 5A**). In addition, APLN-17 at the concentrations of 10^-8^ M (without ML221; **Figure 5C**) and 10^-6^ (W/O ML221; **Figures 5C, D**) increased the protein and mRNA levels of StAR.

### Effect of APLN on IGF1- and FSH-Stimulated E2 and P4 Secretion

The effects of APLN-13/-17 (10^-9^M) in combination with IGF1 (10^-8^M) and FSH (10^-8^M) W/O preincubation of buffalo GCs by ML221 on the secretion of E2 and P4 are presented in **Figure 6**. The results showed that both APLN-13 and APLN-17 in the presence of IGF1 or FSH increased the concentration of E2 (**Figures 6A, B** **)**. Preincubation of the cells for an hour with ML221 decreased the concentrations of E2 and P4 significantly. In addition, both APLN-13 and APLN-17 in the presence of IGF1 or FSH had a significant effect on the levels of P4 W/O ML221 in buffalo GCs (**Figures 6C, D** **)**.

### Effect of APLN Treatment on E2 and P4 Secretion by Buffalo GCs in Response to Various Times of Stimulation by IGF1 and FSH

The effects of APLN-13/-17 (10^-9^ M) on the secretion of E2 and P4 only or in response to various times of stimulation by IGF1 and FSH W/O preincubation of cells with ML221 for an hour are shown in **Figures 7**, **8**. As the results showed, APLN-13/-17 in the presence of IGF1 W/O ML221 had the highest effect on the concentrations of E2 after 24, 48, and 72 h of stimulation (**Figure 7**). APLN-17+FSH had a meaningfully higher effect on the E2 production after 24, 48, and 72 h than APLN-17 W/O ML221 (**Figures 7C, D** **)**. APLN-13 (without ML221; **Figure 8A**) and APLN-17 (W/O ML221; **Figures 8C, D** **)** had a substantially greater effect on the secretion of P4 than their combination with IGF1 or FSH. Compared to APLN-17 or APLN-17+FSH, APLN-17+IGF1 had the greatest impact on P4 secretion at all times of stimulation (24, 48, and 72 h). Furthermore, the secretion of E2 and P4 was diminished while the buffalo GCs were preincubated with ML221 for an hour. In addition, the differences in E2 or P4 production were not statistically different after 48- and 72-h stimulation of cells by different treatments.

## Discussion

**Figure 9:**
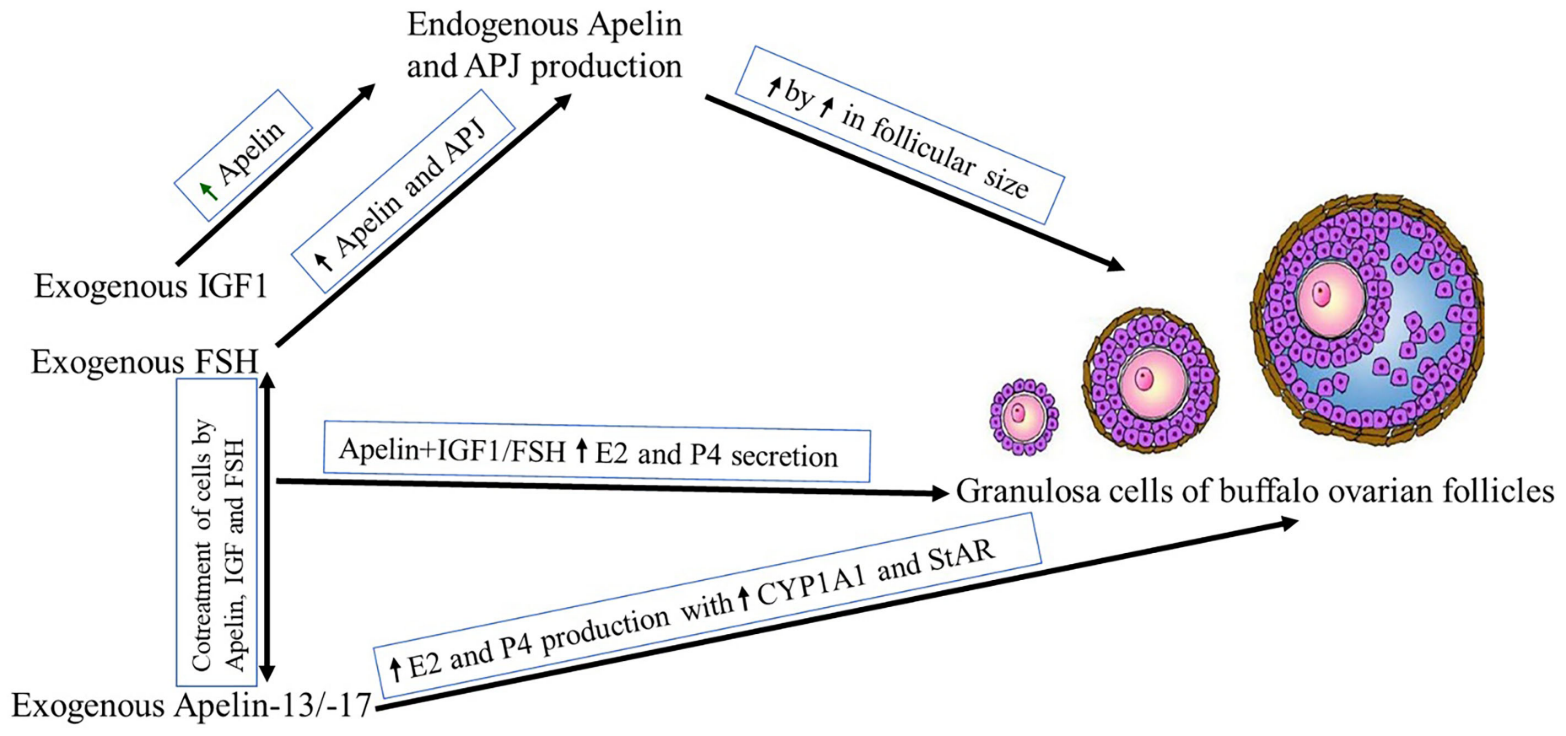
Schematic presentation of the study results. ↑ Stands for the increasing effect.

In the current study, the presence of APLN and its receptor (APJ) was evaluated in GCs of buffalo ovarian follicles with different sizes and the effects of IGF1 and FSH as relevant hormones affecting proliferation and differentiation of ovarian cells, on the expression of APLN/APJ were studied. In addition, the roles of different isoforms of APLN on the E2 and P4 secretion in GCs were investigated (The summarized results are represented in **Figure 9** schematically.). Although the *in vitro* effect of APLN on GCs has previously been declared in various animal species, the protein and mRNA transcript abundances of APLN and its effects on steroidogenesis in GCs have not been addressed in buffalo.

Our results demonstrated that the protein and mRNA of APLN and APJ were expressed in GCs of SF, MF, and LF of buffaloes. The expression of APJ was increased by enlargement of the follicles; however, the expression abundance of APLN was the same in GCs of SF and MF and increased in those of LFs. These results indicated that by the follicular enlargement, the protein expression of APLN significantly enhanced and the highest level of APLN was observed in LFs, whereas the greatest levels of APJ were seen in MFs and LFs. Therefore, the expression of APLN and APJ depends on the ovarian follicle sizes. Similar results were reported by Roche etal. (16) in GCs and oocytes of bovine ovarian follicles even though, unlike our results, the expression of APLN was increased with follicular growth in GCs and oocytes. Likewise, the protein and mRNA transcript levels of APLN/APJ significantly elevated with the follicle augmenting in porcine ovaries (15). Furthermore, Shimizu etal. (12) demonstrated that APLN was not expressed in GCs of bovine ovarian follicles but they showed that APJ mRNA expression increased in line with follicular growth. In other studies, APLN and APJ expressions were detected in GCs of ovarian follicles in sheep (17), pigs (15), and humans (14). The synchronic expression of APLN and APJ in the GCs and other cells of ovarian follicles powerfully implies that APLN may have some paracrine or autocrine roles as a local regulator in GCs, theca cells, and the corpus luteum. The triggering effect of APLN has already been reported on proliferation, migration, and angiogenesis of cells (5, 8, 20, 21); therefore, it can be one of the key factors that have physiological functions in the maturation of follicles. Moreover, according to the reports regarding their expression in different stages of the corpus luteum in porcine (13) and bovine (22), it also influences different actions in the corpus luteum such as vascular formation, maturation, and maintenance (15).

Additionally, hormonal regulation and endogenous hormones can also associate with differences in APLN/APJ expressions in follicles of different sizes because the ovarian conditions change during the ovarian cycle (15).

Furthermore, our results showed that treatment of buffalo GCs with IGF1 at levels of 10^-9^, 10^-8^, and 10^-6^ increased the transcript abundance of APLN; however, FSH only at the dose of 10^-6^ enhanced the expression amounts of APLN or APJ. IGF1 did not change the expression levels of APJ in buffalo GCs. Literature shows that the expression of APLN as an adipokine is regulated by various factors in adipose tissue in humans and mice (23, 24). Nonetheless, the reports regarding the effect of different factors on the APLN/APJ expression levels in GCs are contradictory. Roche etal. (14) reported that FSH/LH had no effect on the expression of APLN and APJ, but IGF1 increased the transcript abundance of APJ in human granulosa cells. Shimizu etal. (12) revealed that P4 elevated the mRNA transcript abundance of APJ in the cultured GCs of cattle, and similar to our results, they suggested that FSH triggered the mRNA expression levels of APJ in the bovine GCs. In another study, Roche etal. (16) demonstrated that IGF1 led to an increase in APLN mRNA expression in bovine GCs whereas it diminished the mRNA expression of APJ. These authors also did not find any significant influence of FSH on the mRNA expression amounts of APLN/APJ. Along with some discrepancies with the literature, our findings revealed that APLN in combination with IGF1 and FSH has a positive effect on steroidogenesis in GCs. It shows that these hormones cooperate in the proper functioning of GCs and interactions among them and other hormones secreted from different ovarian cells are meaningful for the appropriate action of all ovarian cells. However, these inconsistent data can be addressed by some differences in culture condition, type of GCs (from primary, middle, or ovulatory follicles), species, study design, and sample size.

Our results also showed that two isoforms of APLN (-13 and -17) increased the concentrations of E2 and P4 production in buffalo GCs. Higher doses had the greater effects on E2 secretion with preincubation of the cells by ML221 for an hour, but the same effects were not observed without it. Likewise, ML221 decreased both E2 and P4 levels in buffalo GCs treated or untreated by APLN13/-17. Rak etal. (15) found that different doses of APLN increased the production of E2 and P4 in porcine GCs. Similar to our results, Roche etal. (16) suggested that treatment of bovine GCs or human GCs (14) with APLN-13/-17 increased the secretion of P4, and preincubation of cells with ML221 decreased the concentration of P4. There is a disagreement among our findings as they suggested that ML221 did not have any effect on P4 secretion when the GCs were not treated with APLN-13 or APLN-17. On the other hand, APLN-13 and APLN-17 (except 10^-6^ M without ML221) at all doses increased the transcription abundance of CYP19A1. Moreover, APLN-13 with ML221 and APLN-17 without ML221 enhanced the expression of StAR protein in buffalo GCs. In agreement with our findings, CYP19A1 expression levels were improved by treatment of porcine GCs with APLN and it was concurrent along with the increasing effect of APLN on E2 and P4. In contrast to these results, Roche etal. (14) revealed that APLN-13/-17 did not have any meaningful influence on the expression of CYP19A1 or StAR; however, they increased the transcription amount of HSD3B in human cultured GCs. In the current study, the specific effect of APLN on E2 and P4 secretion was correlated with the elevation of StAR and CYP19A1 mRNA levels; accordingly, APLN caused an increase in the production of E2, P4, CYP19A1, and StAR in buffalo GCs. CYP19A1 is an important enzyme for the production of E2, and StAR is a cholesterol carrier required for the preparation of the steroidogenesis precursor. It might be mentioned that an increase in P4 secretion could be connected to an increase in StAR expression abundance and an enhancement in E2 secretion was associated with an enhancement in CYP19A1 expression. Thus, APLN could modulate the activity of StAR and CYP19A1 enzymes in GCs of buffalo ovaries.

Additionally, our results revealed that APLN-13/-17 in combination with IGF1 and FSH increased the concentration of E2 and P4 secretion in buffalo GCs. Likewise, their effects diminished when the cells were preincubated with ML221. The findings also showed that APLN-13 or -17 in the presence of IGF1 substantially had the highest effect on E2 secretion after 24, 48, and 72 h of stimulation. These APLN isoforms also had more effect on the concentration of P4 singly than in the presence of IGF1 or FSH, although APLN-13+FSH with ML221 had a higher effect than APLN-13 or APLN-13+IGF1 in the buffalo GCs. Furthermore, the secretion of E2 and P4 of buffalo GCs was not significantly altered after 48 h of stimulation by different treatments. Roche etal. (16) suggested that APLN elevated E2 and P4 production in human primary GCs and P4 secretion in bovine GCs. These authors also reported that APLN-13 significantly increased P4 secretion W/O IGF1 for 24, 48, and 72 h sequentially, whereas it did not influence the FSH response in line with the time of stimulation in bovine GCs. These results are contrary to our data. In these studies, the P4 concentration increased after 24 h but we did not obtain the same results, and the concentration of P4 and even E2 did not change after 48 or 72 h compared to 24 h of stimulation of buffalo GCs by APLN-13/-17 in combination with IGF1 or FSH. The possible reason to address these discrepancies could be related to species differences and, also, they used primary GCs, whereas we cultivated the GCs from all visible buffalo ovarian follicles. IGF1 is an important endogenous growth factor for the ovaries which have a crucial function in the regulation of follicular development in mammals (25). In such a way, IGF1 triggers GC proliferation and P4 secretion (26). Furthermore, APLN plays a substantial act in energy metabolism and it is an insulin mimic peptide in skeletal muscle. As the structure of insulin and its receptor is similar to IGF1 and IGF1R, APLN could enhance the sensitivity of GCs to IGF1 as already discussed in mouse skeletal muscle; in addition, we discussed the stimulatory effect of APLN on the E2 and P4 secretion, therefore APLN may cooperate with IGF1 or other ovarian hormones in the regulation of steroidogenesis in mammals.

## Conclusions

The results of the current study demonstrated that APLN/APJ is expressed in ovarian GCs of buffalo. A correlation was found between the size of follicles and the expression abundance of APLN and APJ. Our finding also showed that two isoforms of APLN had a stimulatory effect on steroidogenesis and such effect was verified by the specific impact of ML221 as an APJ antagonist. Furthermore, APLN in the presence of IGF1 or FSH, as important hormones influencing the ovarian follicles functions, affected the steroid secretion of buffalo ovarian GCs. The particular effects of different isoforms of APLN on the steroidogenesis of GCs imply that this adipokine has a considerable impact on reproductive functions (especially folliculogenesis) in buffalo (*Bubalus bubalis*), although it needs approval by further studies.

## Data Availability Statement

The original contributions presented in the study are included in the article; further inquiries can be directed to the corresponding author.

## Ethics Statement

The animal study was reviewed and approved by the Animal Ethics Committee of the Guangxi Buffalo Research Institute.

## Author Contributions

BS designed and conducted the study and data analysis and also wrote the manuscript. H-YZ, L-YL, L-PT, X-YM, X-RL, A-QD, YZ, X-HT, C-XH, and Y-YX helped in a few practical lab works. J-HS was the project leader. All authors contributed to the article and approved the submitted version.

## Funding

This study was financially supported by grants from the National Key Research and Development Program (2017YFE0113800), the Key Research and Development Program in Guangxi (GuiKe AB1850013), the Guangxi Natural Science Foundation (2018GXNSFDA050013), and the Special Fund for Guiding Local Scientific and Technological Development by the Central Government (GuiKe ZY21195007) of China.

## Conflict of Interest

The authors declare that the research was conducted in the absence of any commercial or financial relationships that could be construed as a potential conflict of interest.

## Publisher’s Note

All claims expressed in this article are solely those of the authors and do not necessarily represent those of their affiliated organizations, or those of the publisher, the editors and the reviewers. Any product that may be evaluated in this article, or claim that may be made by its manufacturer, is not guaranteed or endorsed by the publisher.

## References

[1] Kershaw EE Flier JS. Adipose Tissue as an Endocrine Organ. J Clin Endocrinol Metab (2004) 89:2548–56. doi: 10.1210/jc.2004-0395 15181022

[2] Pitkin SL Maguire JJ Bonner TI Davenport AP. International Union of Basic and Clinical Pharmacology. LXXIV. Apelin Receptor Nomenclature, Distribution, Pharmacology, and Function. Pharmacol Rev (2010) 62:331–42. doi: 10.1124/pr.110.002949 20605969

[3] Tatemoto K Hosoya M Habata Y Fujii R Kakegawa T Zou M-X. Isolation and Characterization of a Novel Endogenous Peptide Ligand for the Human APJ Receptor. Biochem Biophys Res Commun (1998) 251:471–6. doi: 10.1006/bbrc.1998.9489 9792798

[4] O'dowd BF Heiber M Chan A Heng HH Tsui L-C Kennedy JL. A Human Gene That Shows Identity With the Gene Encoding the Angiotensin Receptor is Located on Chromosome 11. Gene (1993) 136:355–60. doi: 10.1016/0378-1119(93)90495-O 8294032

[5] Shokrollahi B Shang J-H Ahmad HI Yang C-Y Saadati N. Reproductive Roles of Novel Adipokines Apelin, Visfatin, and Irisin in Farm Animals. Theriogenology (2021) 172:178–86. doi: 10.1016/j.theriogenology.2021.06.011 34175524

[6] De Falco M De Luca L Onori N Cavallotti I Artigiano F Esposito V. Apelin Expression in Normal Human Tissues. In Vivo-Athens (2002) 16:333–6. 12494873

[7] Estienne A Bongrani A Reverchon M Ramé C Ducluzeau P-H Froment P. Involvement of Novel Adipokines, Chemerin, Visfatin, Resistin and Apelin in Reproductive Functions in Normal and Pathological Conditions in Humans and Animal Models. Int J Mol Sci (2019) 20:4431. doi: 10.3390/ijms20184431 PMC6769682 31505789

[8] Kasai A Shintani N Oda M Kakuda M Hashimoto H Matsuda T. Apelin is a Novel Angiogenic Factor in Retinal Endothelial Cells. Biochem Biophys Res Commun (2004) 325:395–400. doi: 10.1016/j.bbrc.2004.10.042 15530405

[9] Heinonen M Purhonen A Miettinen P Pääkkönen M Pirinen E Alhava E. Apelin, Orexin-A and Leptin Plasma Levels in Morbid Obesity and Effect of Gastric Banding. Regul Pept (2005) 130:7–13. doi: 10.1016/j.regpep.2005.05.003 15970339

[10] Liu Q Jiang J Shi Y Mo Z Li M. Apelin/Apelin Receptor: A New Therapeutic Target in Polycystic Ovary Syndrome. Life Sci (2020) 260:118310. doi: 10.1016/j.lfs.2020.118310 32835696

[11] Sandal S Tekin S Seker FB Beytur A Vardi N Colak C. The Effects of Intracerebroventricular Infusion of Apelin-13 on Reproductive Function in Male Rats. Neurosci Lett (2015) 602:133–8. doi: 10.1016/j.neulet.2015.06.059 26149233

[12] Shimizu T Kosaka N Murayama C Tetsuka M Miyamoto A. Apelin and APJ Receptor Expression in Granulosa and Theca Cells During Different Stages of Follicular Development in the Bovine Ovary: Involvement of Apoptosis and Hormonal Regulation. Anim Reprod Sci (2009) 116:28–37. doi: 10.1016/j.anireprosci.2009.01.009 19223129

[13] Shirasuna K Shimizu T Sayama K Asahi T Sasaki M Berisha B. Expression and Localization of Apelin and Its Receptor APJ in the Bovine Corpus Luteum During the Estrous Cycle and Prostaglandin F2a-Induced Luteolysis. Reproduction (2008) 135:519–26. doi: 10.1530/REP-07-0409 18367512

[14] Roche J Ramé C Reverchon M Mellouk N Cornuau M Guerif F. Apelin (APLN) and Apelin Receptor (APLNR) in Human Ovary: Expression, Signaling, and Regulation of Steroidogenesis in Primary Human Luteinized Granulosa Cells. Biol Reprod (2016) 95(104):101–12. doi: 10.1095/biolreprod.116.141754 27683264

[15] Rak A Drwal E Rame C Knapczyk-Stwora K Słomczyńska M Dupont J. Expression of Apelin and Apelin Receptor (APJ) in Porcine Ovarian Follicles and *In Vitro* Effect of Apelin on Steroidogenesis and Proliferation Through APJ Activation and Different Signaling Pathways. Theriogenology (2017) 96:126–35. doi: 10.1016/j.theriogenology.2017.04.014 28532828

[16] Roche J Ramé C Reverchon M Mellouk N Rak A Froment P. Apelin (APLN) Regulates Progesterone Secretion and Oocyte Maturation in Bovine Ovarian Cells. Reproduction (2017) 153:589–603. doi: 10.1530/REP-16-0677 28250234

[17] Mercati F Scocco P Maranesi M Acuti G Petrucci L Cocci P. Apelin System Detection in the Reproductive Apparatus of Ewes Grazing on Semi-Natural Pasture. Theriogenology (2019) 139:156–66. doi: 10.1016/j.theriogenology.2019.08.012 31412301

[18] Livak KJ Schmittgen TD. Analysis of Relative Gene Expression Data Using Real-Time Quantitative PCR and the 2– ΔΔCT Method. Methods (2001) 25:402–8. doi: 10.1006/meth.2001.1262 11846609

[19] Thakre A Gupta M Magar S Bahiram K Sardar V Korde J. Transcriptional and Translational Abundance of Visfatin (NAMPT) in Buffalo Ovary During Estrous Cycle and Its *In Vitro* Effect on Steroidogenesis. Domest Anim Endocrinol (2021) 75:106583. doi: 10.1016/j.domaniend.2020.106583 33249344

[20] Cox CM D'agostino SL Miller MK Heimark RL Krieg PA. Apelin, the Ligand for the Endothelial G-Protein-Coupled Receptor, APJ, is a Potent Angiogenic Factor Required for Normal Vascular Development of the Frog Embryo. Dev Biol (2006) 296:177–89. doi: 10.1016/j.ydbio.2006.04.452 16750822

[21] Mughal A O'rourke ST. Vascular Effects of Apelin: Mechanisms and Therapeutic Potential. Pharmacol Ther (2018) 190:139–47. doi: 10.1016/j.pharmthera.2018.05.013 PMC6165679 29807055

[22] Schilffarth S Antoni B Schams D Meyer HH Berisha B. The Expression of Apelin and Its Receptor APJ During Different Physiological Stages in the Bovine Ovary. Int J Biol Sci (2009) 5:344. doi: 10.7150/ijbs.5.344 19461937 PMC2684680

[23] Boucher J Masri B Daviaud D Gesta S Guigné C Mazzucotelli A. Apelin, a Newly Identified Adipokine Up-Regulated by Insulin and Obesity. Endocrinology (2005) 146:1764–71. doi: 10.1210/en.2004-1427 15677759

[24] Wei L Hou X Tatemoto K. Regulation of Apelin mRNA Expression by Insulin and Glucocorticoids in Mouse 3T3-L1 Adipocytes. Regul Pept (2005) 132:27–32. doi: 10.1016/j.regpep.2005.08.003 16137778

[25] Silva J Figueiredo J Van Den Hurk R. Involvement of Growth Hormone (GH) and Insulin-Like Growth Factor (IGF) System in Ovarian Folliculogenesis. Theriogenology (2009) 71:1193–208. doi: 10.1016/j.theriogenology.2008.12.015 19193432

[26] Reverchon M Rame C Bunel A Chen W Froment P Dupont J. VISFATIN (NAMPT) Improves *In Vitro* IGF1-Induced Steroidogenesis and IGF1 Receptor Signaling Through SIRT1 in Bovine Granulosa Cells. Biol Reprod (2016) 94(54):51–13. doi: 10.1095/biolreprod.115.134650 26792944

